# Persistence of behavioral abnormalities following corticosteroid therapy in children with initial episode of idiopathic nephrotic syndrome: a prospective longitudinal observation

**DOI:** 10.1590/2175-8239-JBN-2021-0043

**Published:** 2021-09-17

**Authors:** Parichay Singh, Om P. Mishra, Shashi K. Upadhyay, Rajniti Prasad, Ankur Singh, Abhishek Abhinay, Akash Mishra, Franz Schaefer

**Affiliations:** 1 Banaras Hindu University, Institute of Medical Sciences, Department of Pediatrics, Division of Pediatric Nephrology, Varanasi, India.; 2 Jawaharlal Postgraduate Institute of Medical Education and Research, Department of Biostatistics, Puducherry, India.; 3 Heidelberg University Medical Centre, Centre for Pediatrics and Adolescent Medicine, Division of Pediatric Nephrology, Heidelberg, Germany.

**Keywords:** Child Behavior Disorders, Child, Adrenal Cortex Hormones, Nephrotic Syndrome, Transtornos do Comportamento Infantil, Criança, Corticosteroides, Síndrome Nefrótica

## Abstract

**Introduction:**

Treatment of nephrotic syndrome with corticosteroid can cause several side- effects including behavioral abnormalities. The objectives of the study were to observe the proportion of non-relapsers having persistence of behavioral abnormalities after completion of treatment of initial episode and compare the abnormalities with relapsers, and to determine risk factors for persistence.

**Methods:**

Seventy-five children with a first episode of idiopathic nephrotic syndrome and 60 normal children were rated by parents for behavioral problems using the Child Behavior Checklist. The Parenting Stress Index was also evaluated. The children were rated before treatment and 12 and 36 weeks after.

**Results:**

Both relapsers and non-relapsers showed abnormalities in internalizing and externalizing domains at 12 weeks of steroid therapy. Non-relapsers had abnormal scores in the internalizing domain in 63.5 % and externalizing domain in 48.1% of cases at 36 weeks. Relapsers had abnormal scores in all the three behavior domains, but a significantly higher proportion of relapsers had abnormal scores regarding total behavior (65.2% vs 28.8%, p<0.01) and child domains (100% vs 57.7%, p<0.001) of Parenting Stress Index in comparison to non-relapsers at 36 weeks. Occurrence of relapse increased the risk (odds ratio 5.76, 95% CI 1.35-10.76, p< 0.001) for persistence of abnormal total behavior at 36 weeks follow-up.

**Conclusion:**

Persistence of abnormalities was observed not only in relapsers but also in non-relapsers. Relapse was found to be a significant risk factor for persistence of abnormal behaviors in these patients.

## Resumo

**Introdução:**

O tratamento da síndrome nefrótica com corticosteroide pode causar vários efeitos colaterais, incluindo anormalidades comportamentais. Os objetivos do estudo foram observar a proporção de não-recidivos com persistência de anormalidades comportamentais após conclusão do tratamento do episódio inicial, comparar as anormalidades com os recidivos, e determinar fatores de risco para persistência.

**Métodos:**

75 crianças com primeiro episódio de síndrome nefrótica idiopática e 60 crianças normais foram avaliadas pelos pais por problemas comportamentais usando o Checklist de Comportamento Infantil. O Índice de Estresse Parental também foi avaliado. As crianças foram avaliadas antes do tratamento, 12 e 36 semanas após.

**Resultados:**

Tanto recidivos quanto não recidivos mostraram anormalidades nos domínios de internalização e externalização às 12 semanas de terapia com esteroides. Não-recidivos apresentaram pontuações anormais no domínio de internalização em 63,5%, e no domínio de externalização, em 48,1% dos casos em 36 semanas. Recidivos tiveram pontuações anormais em todos os três domínios de comportamento, mas uma proporção significativamente maior de recidivos apresentou pontuações anormais em relação ao comportamento total (65,2% vs 28,8%, p<0,01) e domínios infantis (100% vs 57,7%, p<0,001) do Índice de Estresse Parental em comparação com não recidivos às 36 semanas. A ocorrência de recidiva aumentou o risco (odds ratio 5,76, 95% IC 1,35-10,76, p< 0,001) de persistência de comportamento total anormal em 36 semanas de acompanhamento.

**Conclusão:**

A persistência de anormalidades foi observada não apenas em recidivos, mas também em não recidivos. A recidiva foi um fator de risco significativo para a persistência de comportamentos anormais nesses pacientes.

## Introduction

Corticosteroid is the mainstay of therapy in children with idiopathic nephrotic syndrome and 90% of the patients are steroid-responsive^1^. About 60-90% of initial steroid responders experience relapsing course requiring repeated courses of steroid therapy^2^^,^ 3. The therapeutic benefits of steroid are accompanied with several side-effects. Attention has been focused on side-effects such as cushingoid facies, growth retardation, cataract, hirsutism, obesity, and behavioral abnormalities^3^^,^ 4.

Using the Child Behavior Checklist (CBCL), some studies have evaluated behavioral abnormalities in children with nephrotic syndrome and found significantly higher scores for different behavioral domains such as anxiety, depression, somatic complaints, aggressive, and hyperactive behavior^5^^-^ 7. Ruth et al. (2004)^8^ reported that the parents of children suffering from nephrotic syndrome rated quality of life as abnormal and family climate, especially maternal stress, negatively affected the behavioral adjustment. Furthermore, it can also affect quality of life in parents in multiple domains of functioning^9^.

Using the CBCL, we have reported behavioral abnormalities in children with idiopathic nephrotic syndrome. Patients of both younger and older age groups had higher scores in most of the domains in comparison to healthy controls^10^. It was also observed that patients in their first episode developed behavioral problems as early as 6 weeks of daily prednisolone^11^. However, whether these behavioral problems persist after discontinuation of treatment of first episode, especially in patients having non-relapsing course who do not require further course of steroid treatment, is unknown.

We hypothesized that behavioral abnormalities should disappear after discontinuation of steroid course in non-relapsers and higher proportion of relapsers may have abnormal behavior scores. Occurrence of relapse, with repeated courses of steroid therapy, can be a risk factor for persistence of abnormalities. Therefore, the present study was undertaken to observe the proportion of non-relapsers having persistence of behavioral abnormalities after completion of treatment of initial episode as primary objective. The secondary objectives were to compare the behavioral abnormalities of non-relapsers and relapsers and identify the risk factors including parental stress for behavioral abnormalities persistence in these patients.

## Materials and Methods

**Figure 1:**
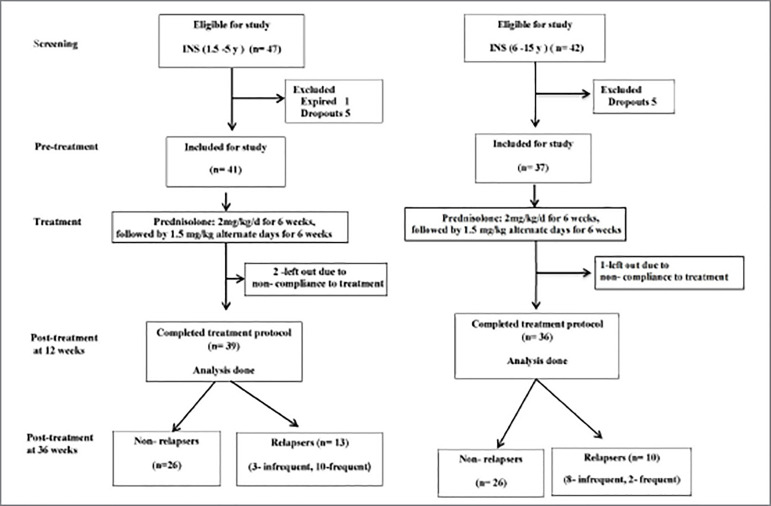
Study flow of patients in 1.5 to 5- and 6 to 15-year age groups (n- number of cases, INS- idiopathic nephrotic syndrome).

The present prospective longitudinal study was conducted at the Division of Pediatric Nephrology, Department of Pediatrics at a tertiary-care center of a teaching hospital. The diagnosis of idiopathic nephrotic syndrome was based on the presence of generalized edema, massive proteinuria (urine protein 3+ or more by Dipstick test and/or spot urine protein/creatinine ratio >2 mg/mg), hypoalbuminemia (serum albumin <2.5 g/dL) and hypercholesterolemia (serum cholesterol >200mg/dL). Patients with gross hematuria, persistent hypertension (systolic and/or diastolic blood pressure above 95^th^ percentile for age, gender, and height on 3 or more occasions), central nervous system infections and vasculitis (diagnosis based on clinical examinations and investigations) and the cases who had received corticosteroid in the preceding period of more than 7 days were excluded. Patients were treated as per the Indian Pediatric Nephrology Group guidelines with prednisolone at a dose of 2 mg/kg/d (maximum 60 mg) in a single dose given for 6 weeks, followed by 1.5 mg/kg (maximum 40 mg) on alternate days for next 6 weeks^12^. The study flow is shown in Figure 1. Assuming the prevalence of behavioral problems following steroid therapy as 68% in nephrotic syndrome and 21.6% in normal controls^13^, a sample size of 60 cases was calculated at α of 5% and power (1-β) of 90%, accounting for 20% drop-outs. Since during the study period a higher number of eligible patients were reported, we had 39 in the younger and 36 in the older age groups. The protocol of the study was approved by the Institute Ethical Committee and informed consent was obtained from a parent (mother/father) of each child.

The Achenbach system of empirically based assessment (ASEBA) was used to rate the child's behavior with application of the CBCL as a tool 14, which has been widely used previously and has cross-cultural validation also^15^^,^ 16. Healthy children who came to the pediatric outpatient department for routine health check-up in each age group were also included as control participants. Different CBCL scales were used for two different age groups (1.5 to 5 years and 6-15 years). The CBCL tool comprises 100 items for younger and 113 items for older age group of children. The respondents were asked to rate their child's behavior using a three-point Likert scale of 0 (not true), 1 (somewhat or sometimes true), and 2 (very true or often true). In the younger age group, behavioral domains are described as emotionally reactive, anxious/depressed, somatic complaints, withdrawn, attention problems, and aggressive behavior. The behavioral domains for the 6-15-year group were anxious/depressed, withdrawn/depressed, somatic complaints, social problems, thought problems, attention problems, rule-breaking behavior, and aggressive behavior. Parents were not informed about the change in scores of the child's behavior following steroid treatment during the study period. The patients were rated for their behavior when they were free of any infections or dysnatremia (hyponatremia or hypernatremia). While completing the questionnaire, the parents were asked to rate their child behavior in the preceding month before starting therapy at the time of enrolment to obtain baseline data, and the tool was again administered by asking the same parent to complete the CBCL at 12-week and 36-week follow-up. The total raw score for each measurement was obtained by adding the scores of all the individual items for a particular domain. These raw scores were converted into normalized 'T' scores. The individual behavioral domains were further sub-grouped into broad group of syndromes as internalizing and externalizing behavior problems in both age groups. The total behavior problem score was obtained by adding the scores of the internalizing and externalizing domains. The T score of <60 was considered normal, between 60 to 63, borderline, and more than 63 as clinical abnormality for internalizing, externalizing and total behavior problems.

The Parenting Stress Index (PSI) assessment tool 17 has 101 general items (46 items in child domains and 55 items in parent domains) and 19 additional optional life stress items, which assesse stressful parent-child interaction. The Child domains are distraction/hyperactivity, reinforcement, mood, adaptability, acceptability, and demandingness, while parental domains include sense of competence, attachment, depression, role of restrictions, relationship with spouse, social isolation, and parent health. Each question was rated by respondents on a 5-point Likert scale: 1 (strongly agree), 2 (agree), 3 (not sure), 4 (disagree), and 5 (strongly disagree). Parents (mother/father) were asked to respond to items pertaining to each domain. Total scores in each group were calculated on the provided scoring sheet, thereafter scores were summarized and converted into percentage. Scoring of parenting stress either of the child's domain or the parent's domain was considered high with scores ≥60% and low with scores <60%. The PSI was also administered thrice, i.e., at pre-treatment, at 12 weeks of completion of prednisolone therapy, and at 24 weeks after completion (i.e., at 36 weeks follow-up). The socioeconomic status of the family was recorded by the modified Kuppuswamy scale for urban^18^ and revised scale for rural population^19^.

### Statistical Analysis

The data were analyzed using SPSS software version 19.0. Socioeconomic status was grouped into upper, upper middle with lower middle, and upper lower with lower in order to have adequate number of cases in each group for analysis. Chi-square and Fisher's exact tests were applied to compare the categorical variables. Student's t-test was used to compare the data between controls and pre-treatment group. Repeated measures analysis of variance (ANOVA) was used for comparison of variables among pre-treatment, 12 weeks, and 36 weeks. Bonferroni test was applied as post-hoc analysis for comparison between each two groups. Univariate and multiple regression analyses were performed to identify the risk factors affecting the behavioral domains. A p-value of <0.05 was considered as significant.

## Results

Seventy-five children with first episode of idiopathic nephrotic syndrome [39 cases aged 1.5 to 5 years (26 non-relapsers and 13 relapsers) and 36 cases aged 6 to 15 years (26 non-relapsers and 10 relapsers)] and 60 normal children (30 in each age group) were included. There were 45 male and 30 female patients and 37 males and 23 females in controls. All patients had normal renal function tests and were steroid-responders.

The mean T scores of different CBCL domains in 1.5 to 5 years and 6 to 15 years age groups are presented in Tables 1 and 2, respectively. Patients had significantly higher mean scores in domains of anxious/depressed and somatic complaints in the younger age group in comparison to controls. Mean scores in children of the younger group in all the behavioral domains, except sleep and somatic problems, were significantly higher in post-treatment at 12 weeks. In the older age group, significantly higher scores of anxious/depressed, thought problems, rule breaking behavior, and aggressive behavioral domains were found at 12 weeks post treatment. Emotionally reactive and anxious/depressed in younger and rule breaking and aggressive behavior in older children showed significant increase at 36 weeks in comparison to their pre-treatment values.

**Table 1:** CBCL T scores in controls and non-relapsers aged 1.5-5 years (mean ±SD)

Behavioral domains	(A) Controls (n=30)	(B) Pre-treatment (n=26)	(C) Post-treatment at 12 weeks (n=26)	(D) Post -treatment at 36 weeks (n=26)	p
Emotionally reactive	51.33	51.77	62.92***	61.73***	
	±1.60	±2.06	±3.61	±3.23	<0.001
Anxious/ depressed	52.23	53.50*	66.00***	64.62***	
	±1.59	±2.37	±3.29	±2.98	<0.001
Somatic complaints	56.17	58.50*	58.65	57.81	
	±3.05	±3.67	±3.60	±3.67	0.611
Withdrawn/ depressed	56.50	57.27	59.73**	58.27	
	±2.92	±2.84	±3.03	±2.22	<0.01
Sleep problems	51.87	52.00	52.96	52.92	
	±1.98	±2.37	±2.97	±2.58	0.293
Attention problems	53.60	53.19	56.42**	54.73	
	±2.84	±2.61	±3.81	±2.91	<0.01
Aggressive behavior	54.40	54.27	60.92***	60.08***	
	±2.40	±2.62	±2.51	±3.21	<0.001

n: number of cases. A vs B:

* P< 0.05; B vs C:

** P<0.01,

***P<0.001; B vs D: ***P <0.001.

**Table 2:** CBCL T scores in controls and non-relapsers aged 6-15 years (mean ±SD)

Behavioral domains	Controls (n=30)	(B) Pre-treatment (n=26)	(C) Post-treatment at 12 weeks (n=26)	(D) Post-treatment at 36 weeks (n=26)	p
Anxious/ depressed	54.43	53.65	56.38**	55.31*	
	±2.62	±2.37	±2.28	±2.62	<0.001
Withdrawn/ depressed	56.43	55.85	56.69	55.96	
	±3.16	±2.36	±2.15	±2.13	0.289
Somatic complaints	58.90	58.23	59.50	57.46	
	±3.57	±3.36	±3.20	±3.73	0.110
Social problems	54.80	54.69	56.12	55.58	
	±2.51	±2.91	±2.44	±2.18	0.164
Thought problems	54.03	54.15	55.92*	55.69*	
	±2.77	±2.98	±2.41	±2.72	<0.05
Attention problems	53.57	52.88	53.46	53.19	
	±1.57	±1.07	±1.39	±1.63	0.328
Rule breaking behavior	56.93	56.46	60.88**	59.31**	
	±3.19	±2.66	±2.44	±2.89	
Aggressive behavior	55.47	55.04	60.8**	59.92**	<0.001
	±1.99	±2.01	±2.33	±2.35	

n- number of cases, p values between: B vs C *<0.05, **<0.001; B vs D * <0.05, **<0.001

Comparisons of various characteristics between non-relapsers and relapsers are presented in Table 3. Mean cumulative dose of steroid and proportion of cases with high child domains of PSI (≥ 60) at 36 weeks were significantly higher in relapsers as compared to non-relapsers (p<0.001) while other parameters were comparable between the two groups.

**Table 3:** Comparison of various characteristics between non-relapsers and relapsers (n: number of cases)

Characteristics	Non-relapsers (n= 52)		Relapsers (n= 23)			p
Age group (years)	n	%	n		%	
1.5-5	26	50	13		56.5	0.602
6-15	26	50	10		43.5	
Gender						
Male	34	65.4	11		47.8	0.152
Female	18	34.6	12		52.2	
Residence						
Rural	38	73.1	20		87.0	0.186
Urban	14	26.9	03		13.0	
Socioeconomic status						
Upper	5	9.6	2		8.7	0.953
Middle	39	75.0	18		78.3	
Lower	8	15.4	3		13.0	
Child domains at 12 weeks						
High ≥60%	35	67.3	17		73.9	0.567
Low <60%	17	32.7	6		26.1	
Parent domains at 12 weeks						
High ≥60%	7	13.5	0		0.0	0.093
Low <60%	45	86.5	23		100	
Child domains at 36 weeks						
High ≥60%	30	57.7	23		100	<0.001
Low <60%	22	42.3	0		0.0	
Parent domains at 36 weeks						
High ≥60%	8	15.4	6		26.1	0.273
Low <60%	44	84.6	17		73.9	
Hemoglobin (g/dL)	11.5 ±1.5		11.7± 1.7			0.836
Systolic BP (mm Hg)	98.5 ±10.4		96.3± 9.6			0.410
Diastolic BP (mm Hg)	61.2 ±7.9		59.9± 7.8			0.508
Cumulative steroid dose (mg/m^2^)	2937.3 ± 303.2		4095.2±.440.3			<0.001

**Figure 2:**
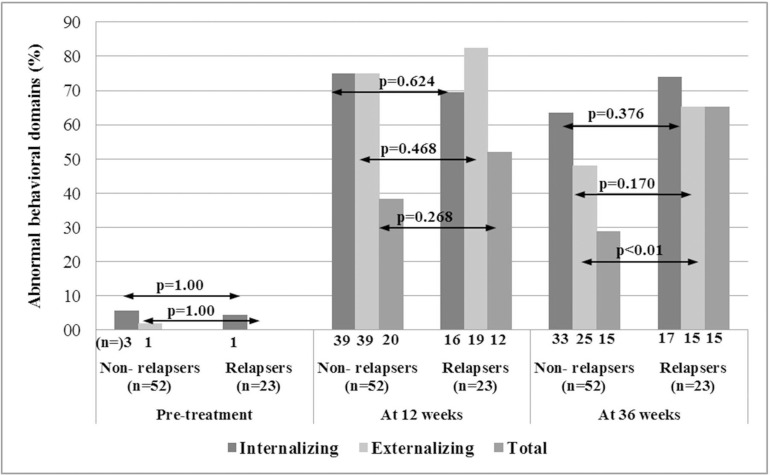
Bar diagram showing percentage of abnormal behavioral domains in non-relapsers and relapsers at pre-treatment, 12 weeks and 36 weeks follow up (n- number of cases).

As per CBCL tool in the younger age group, emotionally reactive, anxious/depressed, somatic complaints, withdrawn/depressed, and sleep problems were grouped into the internalizing domain and attention problems and aggressive behavior in the externalizing behavior domain. Similarly, anxious/depressed, withdrawn/depressed, somatic complaints, social problems, thought problems, and attention problems were categorized into the internalizing domain and rule breaking and aggressive behaviors into the externalizing domain in the older age group. The proportion of children with abnormal internalizing, externalizing, and total behaviors at 12 weeks was similar between non-relapsers and relapsers. At 36 weeks, non-relapsers had abnormal internalizing behavior in 63.5% and externalizing behavior in 48.1% of cases, while relapsers had abnormalities in all the three domains (score ≥60). Only the total behavior score was significantly higher in relapsers than non-relapsers (65.2 vs 28.8%, p<0.01) (Table 4, Figure 2).

**Table 4:** Comparison of behavioral domains between non- relapsers and relapsers (n: number of cases)

Behavioral domains	Non-relapsers (n= 52)		Relapsers (n= 23)		p
	n	%	n	%	
Internalizing behavior score at pre-treatment					
Abnormal ≥60	3	5.8	1	4.3	
Normal <60	49	94.2	22	95.7	1.000
Externalizing behavior score at pre-treatment					
Abnormal ≥60	1	1.9	0	0	
Normal <60	51	98.1	23	100	1.000
Total behavior score at pre-treatment					
Abnormal ≥60	0	0	0	0	
Normal <60	52	100	23	100	-
Internalizing behavior score at 12 weeks					
Abnormal ≥60	39	75.0	16	69.6	
Normal <60	13	25.0	7	30.4	0.624
Externalizing behavior score at 12 weeks					
Abnormal ≥60	39	75.0	19	82.6	
Normal <60	13	25.0	4	17.4	0.468
Total behavior score at 12 weeks					
Abnormal ≥60	20	38.5	12	52.8	
Normal <60	32	61.5	11	48.2	0.268
Internalizing behavior score at 36 weeks					
Abnormal ≥60	33	63.5	17	73.9	
Normal <60	19	36.5	6	26.1	0.376
Externalizing behavior score at 36 weeks					
Abnormal ≥60	25	48.1	15	65.2	
Normal <60	27	51.9	8	34.8	0.170
Total behavior score at 36 weeks					
Abnormal ≥60	15	28.8	15	65.2	
Normal <60	37	71.2	8	34.8	<0.01

Univariate analysis for different variables in relation to persistence of abnormal internalizing, externalizing, and total behavior at 36 weeks is presented in Table 5. The mean systolic BP differed significantly between abnormal and normal internalizing behavior (p<0.01), while systolic BP (p<0.05) and relapse (p<0.01) were found to have significant relationships with abnormal total behavior. Although cases with abnormal internalizing, externalizing, and total behaviors received higher mean cumulative dose of steroid at 36 weeks, it failed to reach the statistical significance. In multiple regression analysis, only occurrence of relapse had an increased risk [odds ratio (OR) 5.76, 95%CI 1.35-10.76, p< 0.001) for persistence of abnormal total behavior at 36 weeks. Although systolic BP had a significant relationships in univariate analysis, it did not affect internalizing (OR 0.999, 95%CI 0.929-1.073, p=970) and total behavior (OR 1.038, 95%CI 0.961-1.122, p=0.343) in regression analysis.

**Table 5:** Univariate analysis of factors affecting behavioral domains (n: number of cases)

	Internalizing behavior at 36 weeks					Externalizing behavior at 36 weeks					Total behavior at 36 weeks				
Parameters	Abnormal (≥ 60) n=50		Normal (<60) n=25		P	Abnormal (≥ 60) n=40		Normal (<60) n= 35		p	Abnormal (≥ 60)n=30		Normal (<60) n=45		p
	n	%	n	%		n	%	n	%		n	%	n	%	
Gender															
Male	29	58	16	64	0.617	22	55	23	65.7	0.345	18	60	27	60	1.000
Female	21	42	09	36		18	45	12	34.3		12	40	18	40	
Residence															
Rural	12	24	05	20	0.697	10	25	7	20	0.606	8	26.7	9	20	0.499
Urban	38	76	20	80		30	75	28	80		22	73.3	36	80	
Relapse status															
Relapser	17	34	06	24	0.376	15	37.5	8	22.9	0.170	15	50	8	17.8	<0.01
Non-Relapser	33	66	19	76		25	62.5	27	77.1		15	50	37	82.2	
Child															
domains															
Abnormal	34	68	18	72	0.723	29	72.5	23	65.7	0.525	19	63.3	33	73.3	0.358
Normal	16	32	07	28		11	27.5	12	34.3		11	36.7	12	26.7	
Parent															
domains															
Abnormal	05	10.0	02	8	1.000	3	7.5	4	11.4	0.853	3	10	4	8.9	1.000
Normal	45	90.0	23	92		37	92.5	31	88.6		27	90	41	91.1	
Socioeconomic status															
Upper	5	10	2	8	1.000	6	15	1	2. 8	0.113	4	13.3	3	6.7	0.427
Middle	34	68	23	92		28	70	29	42.9		197	63.3	38	84.4	
Lower	11	22	0	0		6	15	5	14.3			23.3	4	8.9	
Hemoglobin (g/dL)	11.6 ±1.6		11.4±1.6		0.383	11.4±1.5		11.9±1.6		0.185	11.4± 1.6		11.7± 1.6		0.467
Systolic blood															
pressure (mmHg)	102.7±6.9		95.7±10.9		<0.01	98.5±10.1		96.9±10.2		0.506	100.0± 10.1		94.4±9.3		<0.05
Cumulative															
steroid dose	3481.3		3197.9		0.070	3389.9		3310.9		0.160	3310.9		3280.0		0.840
at 36 weeks (mg/m^2^)	±608.4		±640.7			±652.2		±616.8			±646.7		±643.0		

## Discussion

Patients of nephrotic syndrome can develop behavioral abnormalities following steroid therapy^6^^,^ 10 ^,^ 11. Overall non-relapsers of both age groups showed increase in mean scores in most of the behavioral domains at 12 weeks post-steroid treatment and persisted until the end of follow-up (i.e., 36 weeks). Our previous observations^10^^,^ 11 was limited to 12 weeks of treatment, so no further comment was possible regarding status of resolution or persistence of behavioral abnormalities. In addition, patients of the younger age group had significantly higher mean scores in anxious/depressed and somatic domains at pre-treatment period than controls, and anxious/depressed behavior increased further following steroid therapy, while it was not seen in older children. Soliday et al. (1999)^6^ reported abnormal scores for anxiety/depression and aggressive behaviors at baseline in two children in the younger age group, which also worsened following steroid treatment. It appears that they might be more sensitive in their perception and develop anxiety, depression, or aggression due to physical discomfort with generalized edema before start of the treatment.

Further analysis of different demographic factors showed that a significantly higher proportion of relapsers had abnormal scores in child domains of PSI and received higher mean cumulative dose of steroid. This may be because patients experiencing relapsing courses received additional doses of steroid, affecting PSI in the child's domain. Mehta et al. (1995)^20^ reported a significant relationship of CBCL score with anxiety score of mothers. By contrast, the parent domain of PSI was unaffected in our study. The other possible risk factors which could affect the behavioral scores such as gender, place of residence (rural/urban), socioeconomic status, PSI, hemoglobin level, and cumulative steroid dose were not significant in our study. It may be possible that these factors are not associated with change in their behavior.

The proportion of cases with abnormal internalizing and externalizing behaviors between non-relapsers and relapsers at 12 weeks was similar. Relapsers exhibited abnormalities in all the three domains at 36 weeks, but significantly higher proportion than non-relapsers in their total behavior only (p<0.01). However, persistence of abnormal internalizing behavior in nearly sixty-three percent and externalizing behavior in about half of non-relapsers at 36 weeks is a matter of concern despite the fact that they did not receive any course of steroid during follow-up. It may be possible that non-relapsers still take longer time for resolution of their behavioral problems.

The univariate analysis demonstrated a relationship of systolic BP with internalizing and total behavior domains, but this effect disappeared in multiple regression analysis. Although the relapsers received higher mean cumulative dose of steroid than non-relapsers, no significant association was found with persistence of abnormal behavioral domains in regression analysis. Conversely in our previous study^10^, which also included relapsers and steroid-dependent nephrotic patients, a significant association of cumulative dose of steroid with behavioral abnormalities was found. Youssef et al. (2013)^21^ also found significant correlations of prednisolone dose with mean anxiety, depression, and aggression scores in relapsing nephrotic syndrome using the Child Depression Inventory and the Anxiety Scale for children. Further, in the present study, the occurrence of relapse was found as a significant risk factor for persistence of abnormal behaviors at 36 weeks. Soliday et al. (1999)^6^ had reported increased CBCL scores for anxious/depressed behavior and/or aggressive behavior during relapses in about 70% of children and significantly correlated with steroid dose. However, Ghobrial et al. (2013)^22^ reported that steroid dose and number of relapses did not affect psychological scores in their patients. Thus, it appears that the relationship of steroid dose with behavioral problems is not a straight forward phenomenon. Maybe the relapse during the course of illness can cause more stress and persistence of behavioral abnormalities in these children. In addition, it may be possible that some other factor, like parents being over-concerned about their child illness, affects the child's behavior^7^^,^ 20. However, we did not find any influence of Parenting Stress Index parameters (child and parent domains) over persistence of abnormal behaviors in the present study.

The strength of the present study was the adequate number of cases of both age groups, a long follow- up until 24 weeks after treatment of initial episode, and using a validated tool to rate child's behavior, which depicted almost a true picture of behavioral problems in these children. However, the study has certain limitations, such as the findings being based on parent's reported observations and the control participants being assessed only one time, therefore their further status cannot be commented on. In addition, some of the younger patients had already abnormal rating of behavior (anxious/depressed and somatic domains) in the pre-treatment period, which got aggravated further in the follow-up.

In conclusion, behavioral abnormalities can persist in non-relapsers also but relapsers showed abnormal behaviors in higher proportion of cases. The occurrence of relapse was found as a significant risk factor for persistence of behavioral problems in children with nephrotic syndrome. A long-term follow up regarding the resolution of problems in non-relapsers is recommended.
